# New approaches in developing medicinal herbs databases

**DOI:** 10.1093/database/baac110

**Published:** 2023-01-10

**Authors:** Zahra Fathifar, Leila R Kalankesh, Alireza Ostadrahimi, Reza Ferdousi

**Affiliations:** Department of Health Information Technology, School of Management and Medical Informatics, Tabriz University of Medical Sciences, Daneshgah St., Tabriz 5165665811, Iran; Department of Health Information Technology, School of Management and Medical Informatics, Tabriz University of Medical Sciences, Daneshgah St., Tabriz 5165665811, Iran; Nutrition Research Center, Department of Clinical Nutrition, School of Nutrition and Food Sciences, Tabriz University of Medical Sciences, Tabriz /Ave. Golghast Atakar Neyshabouri, Tabriz 5166614711, Iran; Department of Health Information Technology, School of Management and Medical Informatics, Tabriz University of Medical Sciences, Daneshgah St., Tabriz 5165665811, Iran

## Abstract

Medicinal herbs databases have become a crucial part of organizing new scientific literature generated in medicinal herbs field, as well as new drug discoveries in the information era. The aim of this review was to track the current status of medicinal herbs databases. Search for finding medicinal herbs databases was carried out via Google and PubMed. PubMed was searched for papers introducing medicinal herbs databases by the recruited search strategy. Papers with an active database on the web were included in the review. Google was also searched for medicinal herbs databases. Both retrieved papers and databases were reviewed by the authors. In this review, the current status of 25 medicinal herbs databases was reviewed, and the important characteristics of databases were mentioned. The reviewed databases had a great variety in terms of characteristics and functions. Finally, some recommendations for the efficient development of medicinal herbs databases were suggested. Although contemporary medicinal herbs databases represent much useful information, adding some features to these databases could assist them to have better functionality. This work may not cover all the necessary information, but we hope that our review can provide readers with fundamental concepts, perspectives and suggestions for constructing more useful databases.

## Introduction

Historically, medicinal herbs were used in various modern medical systems and traditional medical systems to prevent or treat diseases. Based on historical evidence, ∼60 000 years ago, plants have been used as drugs (1, 2). Since ancient times, people used medicinal herbs to treat their diseases and they looked for cure in the nature (3), recently, the use of natural medicine as a complementary and alternative medicine is increasing around the world, including in developed countries (4–7). Statistics show that ∼80% of the world population uses medicinal herbs or other natural products for their diseases (8). The prevalence of herbal medicine uses varies widely (6–48%) in European Union countries (6). In some countries, phytomedicine or herbal medicine is a part of the health-care systems (9, 10). In Germany, herbal medicine is known as one of the five main elements of classic naturopathy (phytotherapy, hydrotherapy, exercise therapy, dietetic therapy and ‘lifestyle regulation’ therapy) (11). Also, diverse groups of health-care professionals, namely, doctors, nurses, pharmacists and nonmedical complementary and alternative medicine practitioners are involved in herbal medicine (11, 12).

In China, herbal medicine has been integrated into the official health-care system, 95% of general hospitals have traditional medicine departments and traditional Chinese medicine (TCM) is used for the treatment of outpatients and inpatients in hospitals (13, 14). Also, in India an acronym for Ayurveda, Yoga and Naturopathy, Unani, Siddha, Sowa-Rigpa and Homeopathy (AYUSH) is a well-organized sector providing health-care services in both public and private sectors. In view of the strength of AYUSH systems in reducing the disease burden, the efforts to promote these systems and merge with conventional medicine are on for the last few decades (15). Effective integration strategies will promote communication and mutual understanding among different medical systems, evaluate medical care in its totality, ensure equitable distribution of resources, provide a training and educational program for both traditional and conventional medicine and finally generate a holistic health-care system (16). However, for the successful mainstreaming, the operational integration in terms of communication, information sharing and cross-referrals between the conventional and AYUSH systems is very important.

Until recently, information about medicinal herbs was limited to journals, manuals and textbooks. Recently, with the spread of scientific databases, a new way has been developed for sharing information on medicinal herbs (17). These databases collect and provide data on medicinal herbs, ingredients, 2D/3D structures of compounds, related target proteins, relevant diseases and metabolic toxicity, which are essential in medicinal herbs research studies for scientists, physicians and pharmacists (18). They support many aspects of biological research, including information about a gene or a protein and complex applications for data analysis. The usefulness of these databases critically depends on the volume of information, its correct interpretation and the regular updating of the content (19).

Modern biomedical databases generally are different in stored amount of data, specialization, functionality and type of access. However, with a few exceptions, all the available databases are voluminous and include complex data from multiple sources (20). Medicinal herbs databases as a group of biomedical databases have similar specifications. A huge amount of information, including taxonomy, common names, location, medicinal uses and used parts, and modern scientific information, including physicochemical properties, ingredients, genomic information, mechanisms of action and more specific parameter about medicinal plants are curated in these databases. The format of the stored information in these databases is often texts or images.

Few review studies have assessed the medicinal herbs databases. Ningthoujam and colleagues (21) in 2012 reviewed the different approaches for storing ethnopharmacological information about medicinal herbs in databases to reach some minimal standards in medicinal plant database development. They reported some challenges related to sharing information in developing herbal databases. Major challenges reported in this study were ethnobotanical issues (e.g. lack of benchmark model, intellectual property rights, multiple taxonomies, conservation strategies and biopiracy) and technical issues (e.g., lack of regular updates, non-disclosure of publishing year, inaccessibility to the website or relocation to other websites, evolution of hardware and software, obsolescence of systems and high cost of system maintenance).

Another review presented an analytical overview of natural product databases, focusing on their strengths, weaknesses and their limitations, as well as trends in building future databases (22). The study by Xie *et al.* has introduced a considerable number of natural product databases (e.g. TCM Database@Taiwan, TCM-ID, CEMTDD, SuperToxic and SuperNatural). Moreover, some suggestions were reported in this study for doing more efficient role by medicinal herbs databases, as a key player for new drug discovery in the future.

Recently, the significant growth of medicinal herbs in drug discovery has led to the construction of many new databases with advanced features and the fall of numerous databases (23). Recent medicinal herbs databases often emphasize more than before on phytochemical and physicochemical properties due to new efforts for drug discovery from herbs (24).

Assessing the current status of medicinal herbs databases and their contents and providing recommendations based on this assessment can be useful for more up-to-date and efficient future design. The aim of this review is to study the current medicinal herbs databases focusing on their unique function and their source of information. Furthermore, we discuss relationships (i.e. relationships between herbs and ingredients/compounds) and trends in building future databases.

## Materials and Methods

Search for finding medicinal herbs databases was carried out via Google and PubMed. A search of published papers introducing medicinal herbs databases was conducted in October 2021. PubMed was searched by the recruited search strategy. Keywords were medicinal herb* database*, medicinal plant* database*, natural medicine database* and herbal medicine database. Google was also searched for medicinal herbs databases. Both retrieved papers and databases were reviewed by the authors. Papers with an active database on the web were included in the review. Inclusion and exclusion criteria for databases are described in Table 1.

**Table 1.** Inclusion and exclusion criteria for medicinal herbs databases

Inclusion criteria	Exclusion criteria
(i) Medicinal herbs databases (ii) English content (iii) Databases have been created after 2010 (iv) Free access databases	(i) Inaccessible databases (ii) Websites no longer available (iii) Databases with dead access link (iv) Medicinal herbs databases without any related medicinal or therapeutic information

Selected databases were analyzed according to content type/source, aim of creation, accessibility, country, focus area, facilities/features, reference of data, statistics, status of the URL, sustainability and updated information.

### Available medicinal herbs databases

The 25 reviewed databases in this study had a great variety in terms of characteristics and functions. Table 2 shows the list of the included medicinal herbs databases sorted based on the date of construction, focusing on their content and features/facilities that databases provide to users.

**Table 2.** Properties of the included medicinal herbs databases

Database (year of launch)	Provided data types and source of data	Database features or facilities	Statistics of database	URL
GreenMolBD: nature-derived bioactive molecule database of Bangladesh (2021) (41)	List of herbs from textbooks Compound descriptors from ClassyFire, PubChem, ChemSpider and ChEMBL Target collection from PubMed, ACS and scholar literature	Basic search Advanced search Browse User’s help Drug Likeliness test	No. of plants: 145 No. of compound: 6837 No. of compound Synonym: 216 280 No. of targets: 1846	https://www.greenmolbd.gov.bd/
HERB (2021) ( 33)	Herbs and ingredients: SymMap, TCM-ID, TCMSP and TCM-ID databases Experiments: GEO References: PubMed, SymMap, HIT, TCMSP and TCMID Targets: PubMed Diseases: PubMed DisGeNet Modern drugs: CMap	Basic search Advanced search Browse User’s help Experiments	No. of herbs: 7263 No. of ingredients: 49 258 No. of experiments: 1037 No. of references: 1966 No. of targets: 12 933 No. of diseases: 28 212 No. of modern drugs: 2837	http://herb.ac.cn
TCMPG (2021) (47)	Herbs’ Latin names and articles about sequenced plants: plaBiPD Species’ information: iPlant Herbs’ information: TCM-ID and HERB Genomic data: scientific literature (PubMed)	Browse Search Tools (including BLAST, JBrowse, SSR Finder, Synteny Viewer and HmmSearch) Visualization	No. of species: 160 No. of genomes: 195 No. of herbs: 255	http://cbcb.cdutcm.edu.cn/TCMPG/
Phytochemdb (2020) (46)	Phytochemicals’ properties: PubChem, ChemSpider, ChEMBL, DNP, and SwissADME web	Search 3D structure of the compound submission	No. of plants: 528 No. of phytochemicals: 8093	https://phytochemdb.com/
SuperTCM (2020) (42)	Plant database: MPNS and NCBI Taxonomy Chemical databases: PubChem and ChEMBL Target-related databases: UniProt, ChEMBL and KEGG Pathway-related databases: KEGG and iPath3.0 Disease-related database: therapeutic target database (TTD)	Search User’s help Common name of herbs in multilanguages	No. of accepted botanical name: 5372 No. of botanical synonyms: 11 794 No. of common plant names: 53 443 No. of herbs: 6516 No. of recipes: 242 No. of ingredients: 55 772 No. of targets: 543 No. of diseases: 8634	http://tcm.charite.de/supertcm
UNaProd (Universal Natural Product Resource) (2020) (3)	Traditional names: Makhzan-al-Advieh Scientific names and drug targets: ChEMBL	Ontology-based Basic search Browse	No. of drugs: 3413 No. of Mizaj^a^: 2044 No. of monographs: 851	https://unaprod.com
MedPServer (2019) (45)	Information regarding plant active constituents, molecular structure, their molecular properties and vendor for purchasing the molecules from scholarly literature such as SciFinder, PubMed, PubChem, books and numerous encyclopedias	Search Browse User’s help Pharmacophore search Vendors’ information	No. of natural products: 1687 (unique no. of molecules: 1124) No. of medicinal plants: 212 No. of vendors’ information: 4448 No. of traditional therapeutic applications: 229	http://bif.uohyd.ac.in/medserver
AromaDb (2018) (17)	Plant details, essential oil name and plant variety: published international literature, CSIR-CIMAP, Lucknow9 essential oils monograph, web link annual reports, and journal (*Journal of Medicinal and Aromatic Plant Sciences*) Secondary and tertiary information such as IUPAC name, chemical class biochemical classes, fragrance type and physical and chemical properties: primary information	Basic search Advanced search Browse Essential oils	No. of plants: 165 No. of aroma molecules: 1321 No. of fragrance type: 358 No. of 3D structures: 1321	http://bioinfo.cimap.res.in/aromadb/
CMAUP (2018) (9)	Plant databases: NCBI TaxonomyDB and Plants of the World Online Chemical databases: PubChem, ZINC and ChEMBL Activity and therapeutical-related databases: UniProt, KEGG, QuickGO, ChEMBL and TTD Literature resources: PubMed and doi.org	Basic search Browse Access plants by location User’s help	No. of useful plants: 5645 No. of medicinal plants: 2567 No. of countries/regions: 79 No. of human target proteins: 646 No. of gene ontology: 2473 No. of KEGG pathways: 234 No. of human diseases: 656	http://bidd.group/CMAUP
ETM-DB: Ethiopian Traditional Medicine Database (2018) (4)	Medicinal herbs and related information: research articles, theses, books and public databases such as NCBI Taxonomy and COCONUT, PubChem and ChemSpider	Basic search Structure search ADMET properties search Physicochemical search Browse	No. of prescription: 573 No. of compound: 4285 No. of phenotype: 5621 No. of gene/protein: 11 621 No. of herb-phenotype: 5779 No. of herb-compound: 16 426 No. of prescription-herb: 1879 No. of prescription-phenotype: 573 No. of compound-phenotype: 105 202 No. of compound-gene/protein: 162 632 No. of phenotype-gene/protein: 16 584	http://biosoft.kaist.ac.kr/etm
IMPPAT (2018) (10)	Phytochemical composition: published databases Medicinal uses: books on Indian traditional medicine and NutriChem database Traditional medicinal formulation: TKDL Structure: Balloon and Open Babel Physicochemical properties: FAF-Drugs4 web server and RDKit	Basic search Advanced search - User’s help	No. of Indian medicinal plants: 1742 No. of phytochemicals: 9596 No. of therapeutic uses: 1124 No. of plant–phytochemical associations: 27 074 No. of plant–therapeutic associations: 11 514 No. of traditional Indian medicinal formulations: 974	https://cb.imsc.res.in/imppat
SymMap (2018) (5)	TCM symptoms and herb terms: Chinese Pharmacopoeia MM symptom: MeSH, SIDER and UMLS Ingredients: TCM-ID, TCMSP and TCM-ID Target component: HIT, TCMSP, HPO, DrugBank and NCBI gene databases Disease: OMIM and Orphanet	Search Browse User’s help	No. of herbs: 698 No. of ingredients: 26 035 No. of targets: 20 965 No. of diseases: 14 086 No. of TCM symptoms: 1148 No. of MM symptoms: 2518 No. of herb–TCM symptom: 6638 No. of TCM symptom–MM symptom: 2978 No. of herb–ingredient: 48 372 No. of MM symptom–disease: 12 107 No. of ingredient–target: 29 370 No. of gene–disease: 7256	http://www.symmap.org
YaTCM (2018) (44)	Relationships: TCM databases, TCM integrated database (TCM-ID), TCMSP and Database@Taiwan, TCMSP and TTD OMIM, ChEMBL, KEGG Prescription: PubMed, formulas of Chinese medicine and Chinese pharmacopoeia	Browse Search similarity search User’s help Systematic network for visualization	No. of herbs: 6220 No. of natural compounds: 47 696 No. of targets: 18 697 (with 3461 therapeutic targets) No. of predicted targets: 1907 No. of pathways: 390 No. of prescriptions: 1813	http://cadd.pharmacy.nankai.edu.cn/yatcm/home
PHCD (2017) (43)	Lists of phytochemical ingredients: scientific journals 3D structures: optimized using the MOPAC2016 program	Basic search Structure search Topological search Physicochemical search	No. of herbs: 312 No. of compound: 5546 No. of references: 992	http://persianherb.com
MPOD (2017) (56)	Genomes and transcriptomes information: scientific literature	Search Some bioinformatics tools such as BLAST, Heatmap and JBrowse	No. of published and unpublished genomes: 160 No. of transcriptomes: 228 No. of pathways: 85 No. of catalytic components: 629 enzymes from 8 major gene families	http://medicinalplants.ynau.edu.cn
MPD3: medicinal plants database for drug designing (2016) (57)	Phytochemicals information from published literature, PubMed and PMC 3D structures from chemical databases like MAPS database, ChemBridge, ChEBI, ChEMBL, PubChem and Zinc	Search Activity search Browse User’s help	No. of phytochemicals: 5022 No. of medicinal plants: 1022 No. of activities: 92 No. of references: 980 No. of targets: >200	https://www.bioinformation.info
VIETHERB (2016) (58)	Data from different sources were retrieved, hard-copied documents and online databases Cay Thuoc (medicinal plants), Tropicos, The Plant List, KNapSAcK, GBIF and ChEBI	Basic search Browse Ontology-based structure	No. of species: 2881 No. of metabolites: 10 887 No. of geographical locations: 458 No. of therapeutic effects: 8046 No. of species–metabolite linkages: 17 602 No. of species–therapeutic effect linkages: 2718 No. of species morphology linkages: 11 943 No. of species-distribution: 16 089	http://vietherb.com.vn
MPDB 2.0 (2014) (34)	Search in PubMed for medicinal and nutritive values of Bangladeshi medicinal plants and active compounds of them and in The Plant List web server http://www.theplantlist.org for synonymous plant scientific names	Basic search Advanced search	No. of family plants: 122 No. of genus: 381 No. of species: 557	https://www.medicinalplantbd.com
PharmDB-K (2014) (59)	ChEMBL, CTD, DCDB, DIP, DrugBank, Entrez Gene Interactions, GAD, MATADOR, MINT, OMIM, SIDER, T3DB, Traditional Knowledge Portal and TTD, six pharmacopoeias and published articles	Search Browse User’s help	No. of traditional Korean medicine: 262 No. of drug: 7462 No. of disease: 4010 No. of protein: 32 367	http://pharmdb-k.org http://biomart.i-pharm.or
TM-MC (2014) (60)	Information on medicinal materials and their chemical compounds from MEDLINE and PMC	Search Browse User’s help Synonyms in multilanguage	No. of chemical compounds: 32 657 No. of medicinal materials: 631 No. of articles: >32 million	http://informatics.kiom.re.kr/compound
AfroDb (2013) (61)	Plants list, chemical structures of compounds and biological activities from literature sources such as journals, theses, textbooks and 3D structures were generated using the MOE software, and ADMET-related properties were calculated by using the QikProp program	Search Browse User’s help Diversity analysis	No. of compounds: 1008	http://african-compounds.org/about/afrodb/
TCM-ID (2012) (62)	Encyclopedia of TCM Molecular structures: pharmacological activities Information for herbs: TCM-ID database Herbal ingredients from TCM@Taiwan, TCM-ID Diseases, proteins, drugs and targets: DrugBank and OMIM	Search	No. of herbs: 10 846 No. of prescriptions: 99 582 No. of ingredients: 43 413 No. of drugs: 8182 No. of diseases: 4633 No. of TCM diseases: 2679 No. of prescription-ingredients: 1045 No. of herbal mass spectra: 778 No. of mass spectrometry of ingredients: 3895	http://www.megabionet.org/tcmid
TCM Systems Pharmacology Database and Analysis Platform (TCMSP) (2012) (7)	Herbs list: Chinese pharmacopoeia (2010), DrugBank, PubChem, PharmGKB, U.S. Food and Drug Administration (FDA) and UniProt	Basic search Advanced search User’s help	No. of herbs: 499 No. of chemicals: 12 144 No. of diseases: 837 No. of drug targets: 3311	https://old.tcmsp-e.com/tcmsp.php
TCM Database@Taiwan (2011) (6)	Herbs list: Chinese medical texts and dictionaries TCM constituents: MEDLINE and ISI Web of Knowledge Structures and properties: ChemBioOffice 2008	Basic search Structure search Physicochemical search Browse	No. of herbs: 453 No. of compounds: 32 364	http://tcm.cmu.edu.tw/

a Mizaj is defined as the uniform quality, created via the interaction of the four qualities constituting a substance in various proportions (hotness, coldness, wetness and dryness are foundational concepts in Iranian traditional medicine).

**Figure 1.**
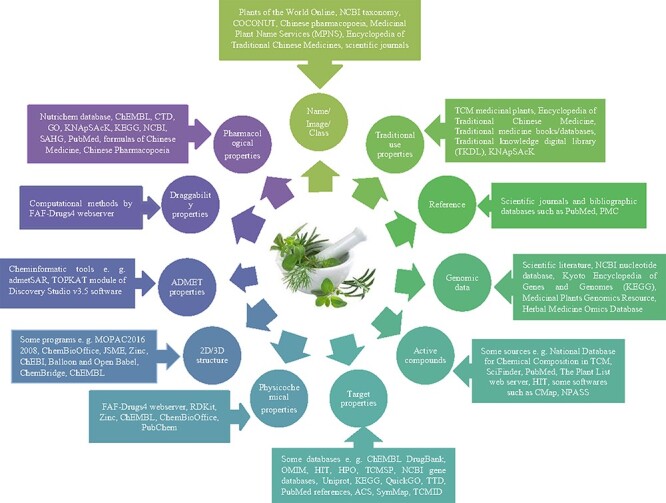
Existing information types and their resources in medicinal herbs databases.

It should be pointed out that more beneficial information could be available in medicinal herbs databases from different sources. Figure 1 demonstrates the possible types of information about medicinal herbs (circles) and their source information (rectangles). As summarized in Figure 1, scientific literature, journals, textbooks, online specialized databases, national pharmacopoeias, traditional medicine resources and some software/programs (computational tools) for performing computational approaches and predicting associations are the common resources and tools that were recruited to construct the medicinal herbs databases.

### Recruited resources in medicinal herbs databases

The data for the creation of a database may be obtained from various sources. All the reported databases in this review provided information about the list of medicinal herbs, their medicinal uses, used part (i.e. root, leaves, seeds, flower and fruit), common names and their synonyms that were collected from various resources (e.g. traditional medicine textbooks/online databases, national pharmacopoeias, scientific journals and National Center for Biotechnology Information (NCBI) Taxonomy). Other characteristics such as physicochemical properties, molecular structures/properties, absorption, distribution, metabolism, excretion and toxicity (ADMET)-related properties and ingredients have been gathered in medicinal herbs databases from computational methods or from existing resources (e.g. online specialized chemical databases such as PubChem (25), ChEBI (26), ChEMBL (27), ChemSpider (28), ChemBioOffice (29), Balloon and Open Babel (30), FAF-Drugs4 web server (31) and RDKit (32)). It is a remarkable point to highlight the importance of segregation between the observed pharmacological properties from wet laboratories and the predicted pharmacological properties from dry laboratories.

### Features of the medicinal herbs databases

The basic characteristics of included databases in this study are summarized in Table 3.

**Table 3.** Characteristics of selected databases

Database	Created in	Last update	Country	Download	Data submission	Plant portrait	Cross ref.	Reference
GreenMolBD	2021	2022	Bangladesh	Y	N	Y	Y (PubChem)	Y
HERB	2021	NA	China	Y	N	N	Y (SymMap, CMap, TCM-ID, TCMSP, GEO, TCM-ID, PubMed, GO, KEGG and PubChem)	Y
TCMPG	2021	NA	China	Y	N	Y	Y (TCM-ID, HERB, SymMap and PubMed)	Y
Phytochemdb	2020	NA	Bangladesh	Y	Y	N	N	N
SuperTCM	2020	NA	Germany	N	N	Y	Y (NCBI Taxonomy browser, ChEMBL, PubChem and KEGG)	N
UNaProd	2020	NA	Iran	N	N	N	Y (IrGO and CMAUP)	N
MedPServer	2019	NA	India	N	Y	Y	N	Y
AromaDb	2018	2020	India	partial	N	Y	Y (PubChem)	N
CMAUP	2018	NA	Singapore	Y	N	Y	Y (ZINC, PubChem, NCBI TaxonomyDB, UniProt, ChEMBL, QuickGO and KEGG)	N
ETM-DB	2018	NA	Ethiopia	N	N	N	Y (PubChem and ChemSpider)	Y
IMPPAT	2018	2022	India	N	N	N	Y (PubMed, InChI Key, The Plant List database, Tropicos and ChemSpider)	Y
KampoDB	2018	2022	Japan	N	N	Y	Y (TradMPD, PubChem, QuickGO and KEGG)	N
SymMap	2018	2021	China	Y	N	N	Y (MalaCards, GeneCards, HERB, OMIM, TCM-ID, TCM-ID, TCMSP, UniProt and GenBank)	Y
YaTCM	2018	NA	China	N	N	Y	Y (KEGG and ChemBL)	N
PHCD	2017	NA	Iran	N	Y	Y	N	Y
MPOD	2017	2021	China	Y	Y	Y	Y (PubMed, NCBI and National Genomic Data Center)	Y
MPD3	2016	NA	Pakistan	Y	N	N	N	Y
VIETHERB	2016	NA	Vietnam	Y	N	Y	Y (PubChem and KNapSAcK)	N
MPDB 2.0	2014	2021	Bangladesh	N	N	N	Y (PubMed)	Y
PharmDB-K	2014	2020	South Korea	N	N	Y	Y (PubChem, PubMed and Gene)	Y
TM-MC	2014	2021	Northeast Asia	Y	N	Y	Y (PubChem and PubMed)	Y
AfroDb	2013	NA	Africa	Y	N	N	Y (PubChem, PubMed, NCBI Taxonomy and reference link)	Y
TCM-ID	2012	2017	China	partial	N	N	Y (UniProt, OMIM and PubChem)	N
TCMSP	2012	2020	China	Y	N	N	Y (FDA, DrugBank, PubChem, UniProt and PharmGKB)	N
TCM Database@Taiwan	2011	NA	China	Partial	Y	N	N	Y

According to the results, all databases mentioned provide their deployment year, but the last updated date of the databases was displayed only in 44% (11 out of 25 databases). Of 25 reviewed databases, 5 claimed that they have facilities for new information submission, but only in three of them [Persian Herbal Constituents Database (PHCD), MedPServer and Phytochemdb], a mechanism was foreseen and a form was provided for submitting new data by registered users. More than half of the databases (52%) provided images of medicinal plants, and 40% of them did not document their information by any reference. Of 25 databases, 20 have cross-references with other scientific databases. Most cross-references were with PubChem (13 out of 25), PubMed (8 out of 25) and KEGG^1^ (5 out of 25). China with eight medicinal herbs databases and Bangladesh and India with three databases have the highest number of databases among the selected databases.

### Cross-referencing in medicinal herbs databases

There were some collective integrated databases included in our study (9, 33, 34), suggesting that studying the features of these databases may be helpful in designing future databases. An integrated database is a collection of data from different sources organized under one structure. The database can create links between the separate data, based on common elements, information or programming logic (35). Due to the quick growth of biomedical information, it seems that there is an urgent need for the development of integrated and collected databases. Integrating the information in databases could lead to time-saving for users because they will have access to the content of more than one database in a database search or browse.

Moreover, for preventing duplicated unnecessary information in databases, information linking/referencing between different databases could be highly helpful, which could be achieved by cross-referencing. Cross-referencing in biomedical databases is highly important and improves the functionality of the databases. Also, cross-referencing could provide networks of related data for a wide range of researchers to use scientific databases. SymMap, CMAUP and HERB were databases with the most cross-referencing to scientific databases. PubMed, PubChem, KEGG and ChEMBL were the most common cross-referenced databases used by medicinal herbs databases. It seems to have a unique ID (identifier) or unified nomenclature, like in UniProt (36) for proteins, which is a necessary element for integrating data from different databases and cross-referencing between databases. A Life Sciences Identifier (LSID) (37), which is a way to name and locate pieces of information on the web, is represented as a uniform resource name (38). Essentially, an LSID is a unique identifier for some data, and the LSID protocol specifies a standard way to locate the data (as well as a standard way of describing that data). LSIDs are a little like Digital Object Identifiers (DOIs). There has been a lot of interest in LSIDs in both the bioinformatics and biodiversity communities (39). However, more recently, as understanding has grown about how HTTP Uniform Resource Identifier can perform a similar naming task, the use of LSIDs as identifiers has been criticized. Alternative identifiers have been proposed for organisms, e.g. the DOI system. NamesforLife (40), a private company, set up a system to apply DOIs to organisms. The potential application of these systems in integrating information about medicinal herbs is expected.

It should be pointed out that the provided links should be updated regularly. In some databases, some provided links had been changed and were not available.

### Future directions for medicinal herbs databases

**Figure 2.**
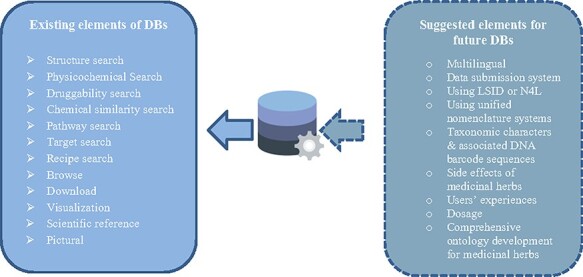
Designing characteristics of medicinal herbs databases.

We mentioned existing elements of medicinal herbs databases in the left rectangle of Figure 2. Databases are designed to enable access to entries by different keywords, including the name of the plant, compound, target and disease by using multiple searches, browsing facilities, visualizing and downloading data. The advanced search options including physicochemical search, druggability search, chemical similarity search and recipe search enable the user to search compounds based on their physicochemical properties, such as molecular weight, XLogP, topological polar surface area, drug-likeness test, and chemical similarity (17, 41). Using the pathway search, users can query in Mapper with KEGG IDs to derive the KEGG pathways that were affected by special herbs and recipes (42).

The other facility is structure search. In some medicinal herbs databases, the advanced search option incorporates a molecular drawing interface for structure search by using an on-screen chemical structure drawing tool (4, 6, 9, 43, 44). In the structure retrieval module, users can build or import a molecular structure and perform a similarity or substructure search. They can also specify structure types, including exact search and substructure search, whichever best describes users’ needs. Furthermore, the structural search option makes it possible to find similar shapes and pharmacophore properties to the user-input molecule (9, 45, 46).

Visualization interface is the other feature that some medicinal herbs databases provide to users. This feature represents descriptive information and relationships with other components using network visualization and tables in a better manner. Also, medicinal herbs databases employed visualization tools to display the associations between medicinal herbs, their ingredients, targets, diseases, etc., in the form of a network in different shapes and colors. Finally, the association network can be downloaded using the available export option in databases (5, 7, 10, 33, 47).

Contemporary biomedical databases include a wide range of information types from various observational and instrumental sources (15). Our review of medicinal herbs databases shows that although medicinal herbs databases provide a high volume of useful information, they have fewer functionality characters and some types of information have been ignored. But, the potential application of taxonomic characters and associated DNA barcode sequences in integrating complex information about medicinal herbs is promising. Such information may be linked with genomic data and plant identifier services such as POWO (48), The Plant List (49) and Taxonomic Name Resolution Service (50). Adding some features may be helpful for making data more valuable for research purposes (as shown in Figure 2).

The first challenge with the content of medicinal herbs databases is managing the high volume of data. Some databases (i.e. national databases or related to specific traditional medicine system databases) have limited data, but generally, most of them are holistic and comprehensive databases. They collected massive amounts of data about herbs, their names (i.e. scientific, common and local), therapeutic effects of herbs, physicochemical properties, their ingredients, 2D/3D structures, scientific references, genomic data of medicinal herbs and different existing/possible associations between elements of a medicinal herbs database. Our study showed medicinal herbs databases recruited advanced programs/facilities for managing this much amount of information. They provided some facilities for retrieving and using database information by browsing, searching, visualizing and downloading partial or whole retrieved information. Due to the different available data in databases, they have provided various search possibilities. Structure search, ADMET properties search, physicochemical properties search, similarity search, activity search, simple sequence repeats (SSRs) search, drug-likeness test and pharmacophore search options are some of the common search options in reviewed databases. SSRs play important roles in herbal medicine variety identification, plant germplasm identification, genetic map construction and genetic diversity analysis (22). To obtain SSRs in the medicinal plants, SSR search was used in the TCM Plant Genome database (TCMPG, http://cbcb.cdutcm.edu.cn/TCMPG). Current facilities in medicinal herbs databases and suggested options for future construction of databases are shown in Figure 2. The suggested elements are based on screening other biomedical databases and people’s opinions represented in some papers (51, 52). The multiplicity of observed data and current limitations may be caused to provide a holistic approach in compiling medicinal dataset.

One of the less attending features in studied herb medicinal databases was the possibility of data submission by the users. Because of discovering new species, experiments by researchers, diversity of herbs in geographical regions/countries, further improving the quality of entries in databases by increasing the amount of experimentally verified data with source attribution and diversity of common/local names for the most of medicinal herbs, data submission may be an important option in medicinal herbs databases like in the case of UniProt for proteins. In reviewed databases, some databases have facilities for registered users to submit new data. They could fill necessary fields in the submission form and then upload related files. In UniProt, researchers are able to add articles that they deem relevant to an entry and provide optional basic annotation by selecting the topics relevant to each paper from a controlled list and/or adding short statements about protein name, function and disease (53). The submission page allows the submission and categorization of submitted information for experimental annotations and displays comprehensive data gathered from other databases for each entry (36, 54).

Although ontology has a great role as a backbone technology for knowledge-based systems (16), this feature has been neglected in developing medicinal herbs databases. Only two databases were ontology-based in this review. Ontologies can be used for data selection, data aggregation, decision support, natural language processing and knowledge discovery in biomedical databases (55). Also, awareness about the role of ontologies in conceptualizing between elements of biomedical databases such as disease, proteins, targets and drugs is growing up. It seems that ontologies should be more considered for modern design-integrated and collected databases.

The other suggestions are about dosage, side effects of medicinal herbs and users’ experience of medicinal herbs use. Providing this volume of information in future medicinal herbs databases as well as a multilingual interface could lead databases more useful for many groups of users.

## Conclusion

Medicinal herbs provide the potential of discovering new drugs from nature. Online databases with their new approaches, contents and constructions are pivotal for achieving this aim. However, studies showed that medicinal herbs databases are diverse in terms of content and data representation. Using common accepted standards for constructing databases (in terms of data elements or minimum dataset and functionality) for diverse usage of them by various group of the users can be helpful for integrating information and demonstrating the complex ethnopharmacological knowledge (21).

This review provides a perspective on the current status of medicinal herbs databases. More useful information can be collected in medicinal herbs databases from different sources (e.g. scientific literature/databases and specialized databases), approaches (e.g. computational methods) and programs/software (e.g. ChemBioOffice and CMap). Users can easily browse, search, download and visualize these data, as well as the relationships between database components, using the convenient interface. Constructing multilingual databases, providing information about medicinal herbs dosage, side effects or different interactions of them, users’ experiments, a comprehensive ontology for medicinal herbs, unique identifier/name via a certified organization/database and finally possibility of submitting data are some recommendations for better functionality of medicinal herbs databases.

Medicinal herbs had been the source of treatment for various human diseases from time immemorial. Interests in herbal-based product frames for the discovery of modern drugs have grown in recent years. However, research on exploring the herbal medicinal systems for modern therapeutics is severely limited due to our incomplete understanding of the therapeutic mechanism of action. Most of the information existing about the use of medicinal herbs in disease treatment and medicine recipes in traditional medicine textbooks are based on human experiences, not based on scientific experiments (e.g. Randomized Control Trials). Existing a comprehensive medicinal herbs database with various modern biomedical database features could pave the way for new herb-based drug discoveries.

## Footnotes

1. Kyoto Encyclopedia of Genes and Genomes
